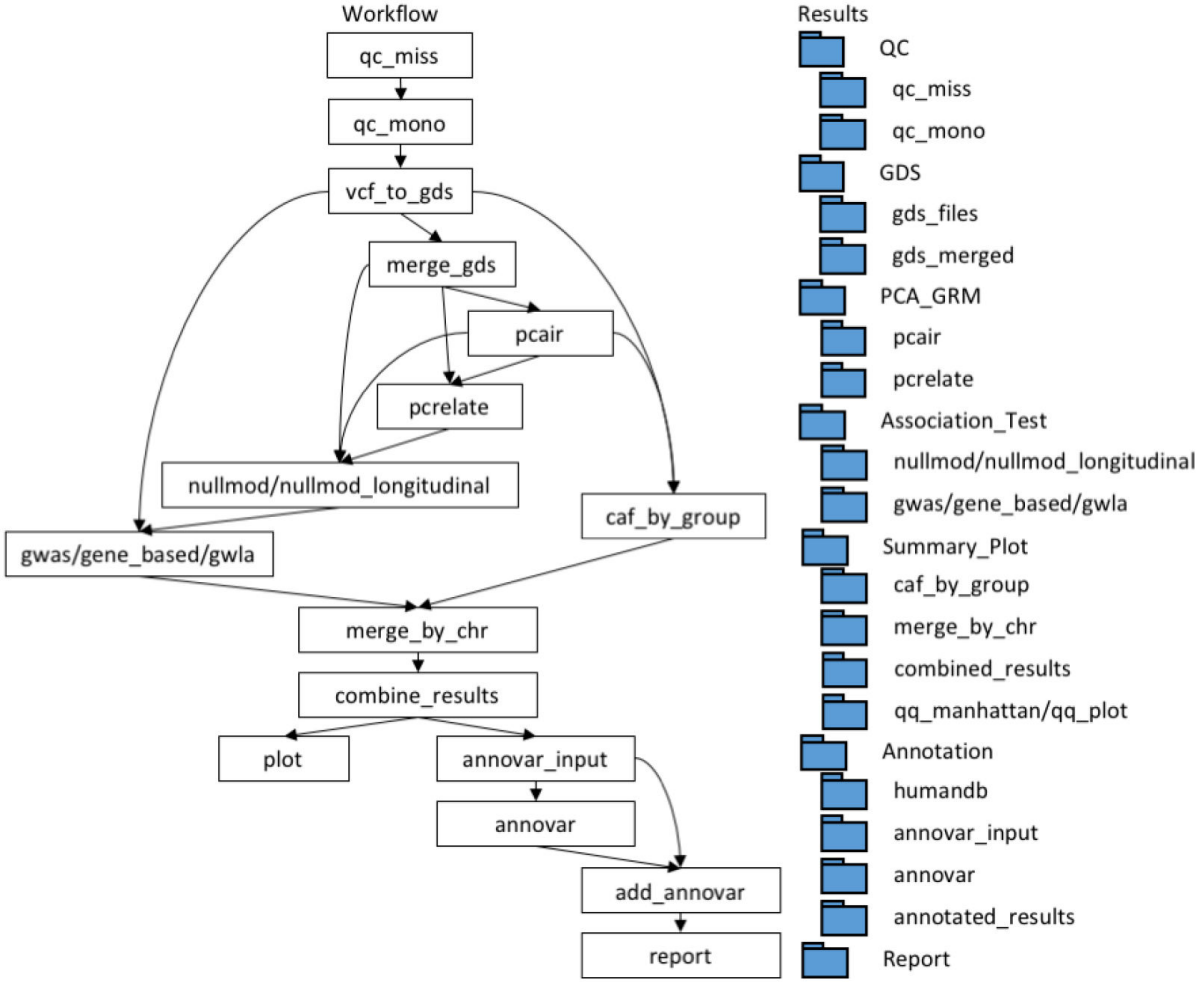# nf-gwas-pipeline: A Nextflow Genome-Wide Association Study Pipeline

**DOI:** 10.21105/joss.02957

**Published:** 2021-03-02

**Authors:** Zeyuan Song, Anastasia Gurinovich, Anthony Federico, Stefano Monti, Paola Sebastiani

**Affiliations:** 1 Department of Biostatistics, Boston University School of Public Health, 801 Massachusetts Avenue 3rd Floor, Boston, MA 02218, USA; 2 Section of Computational Biomedicine, Boston University School of Medicine, 72 East Concord St., Boston, MA 02218, USA; 3 Institute for Clinical Research and Health Policy Studies, Tufts Medical Center, 800 Washington Street, Boston, MA 02111, USA; 4 Bioinformatics Program, Boston University, 24 Cummington Mall, Boston, MA 02215, USA

## Abstract

A tool for conducting Genome-Wide Association Study (GWAS) in a systematic, automated and reproducible manner is overdue. We developed an automated GWAS pipeline by combining multiple analysis tools – including bcftools, vcftools, the R packages SNPRelate/GENESIS/GMMAT and ANNOVAR – through Nextflow, which is a portable, flexible, and reproducible reactive workflow framework for developing pipelines. The GWAS pipeline integrates the steps of data quality control and assessment and genetic association analyses, including analysis of cross-sectional and longitudinal studies with either single variants or gene-based tests, into a unified analysis workflow. The pipeline is implemented in Nextflow, dependencies are distributed through Docker, and the code is publicly available on Github.

## Statement of need

Genome-Wide Association Studies (GWAS) have led to the discovery of highly reproducible variants associated with human complex traits and diseases (Tam V et al., 2019). Many software tools are available for conducting GWAS using cross-sectional data and standard multivariable linear or logistic regression. However, the analysis of longitudinal or family-based studies requires the use of advanced statistical methods such as mixed effect modeling, or survival analysis. To conduct a GWAS with such complex data, often investigators have to rely on multiple programs and ad-hoc linking the results generated between steps because each program focuses on a specific aspect of the analysis (Eller RJ et al., 2019). This approach makes it difficult to keep track of all the analysis steps and parameters, including data cleaning, filtering, and calculation of the genome-wide principal components, limiting reproducibility of the analyses. To address these problems, we have developed a GWAS pipeline that provides a comprehensive computing environment to manage genome-wide genotype data, to conduct analyses of continuous and binary traits using mixed effect models, and to summarize, annotate, and visualize the results.

## Materials and Methods

### Features

The pipeline is implemented in Nextflow, a portable, scalable and parallelizable reactive workflow framework for data-intensive pipelines (Di Tommaso P et al., 2017). Through Nextflow, the GWAS pipeline is designed to automatically parallelize workflow steps, denoted as processes, and does not require users to manually handle intermediate data between steps, thus improving traceability and reproducibility. Additionally, the pipeline remains flexible allowing users to customize parameters and options in the configuration file. Adding processes (i.e., additional analytical steps) in Nextflow framework is standardized and simple. Advanced Nextflow users can define and incorporate additional processes in the pipeline framework, which will then be executed as part of the customized pipeline.

### Workflow

Figure 1 depicts the workflow:

1. Input. Required files include VCF files for all 22 chromosomes and a comma-delimited phenotype file including sample id, phenotype and covariates. Optionally, users can provide a file with a list of Single Nucleotide Polymorphisms (SNPs) to compute genome-wide principal components and genetic relationship or provide a kinship matrix for known relations.
2. Quality control. The pipeline removes monomorphic SNPs and variants with percent of missing values above a provided threshold. The cleaned VCF files are then converted into Genome Data Structure (GDS) file format for computationally efficient data storage (Zheng X et al., 2017).
3. Principal component analysis and genetic relationship inference. This is an optional step that uses the PC-AiR and PC-Relate algorithms (Conomos MP et al., 2015)(Conomos MP et al., 2016) to estimate the genetic relationship matrix between study subjects (GRM) and genome-wide principal components (PCs) to adjust for population structure. Users have the option to provide their own PCs as covariates and/or GRM.
4. Association tests. The pipeline can conduct three types of analyses of qualitative and quantitative traits: GWAS of individual SNPs, gene-based tests, and genome-wide longitudinal analyses, by turning on the corresponding logical options in the configuration file. All three analyses fit a null model first and then iterate over the whole genome by testing one variant or gene at a time. The tests for cross-sectional data are based on the R package GENESIS (Gogarten SM et al., 2019). The test for longitudinal data is based on the R package GMMAT, which uses generalized mixed effect models (Chen H et al., 2016).
5. Output and Visualization. Manhattan and QQ-plots with the value of genomic control are generated for any of the three analyses. The output file includes SNP results annotated with ANNOVAR and minor allele frequency (MAF) for any subgroup of individuals defined by the users. Finally, an html-report of the analysis is generated.

### Configuration file

A single configuration file containing all the pipeline options and parameters is provided. Users can customize this file to deploy specific analysis processes. For example, a user may run a GWAS on a cohort of unrelated individuals by turning off the GRM step. To reduce the complexity for new users, only essential parameters are modifiable in the configuration file. Advanced users can modify any parameters by diving into the original code.

### Installation and Execution

The pipeline can be cloned from https://github.com/montilab/nf-gwas-pipeline. A Docker container was built to assist in installing the necessary tools and the pipeline and ensuring accessibility to specific versions of tools and packages. There are two methods for executing the pipeline. Users can execute the pipeline through the command line interface specifying parameters and a configuration file. All available options can be either obtained by the command ‘–help’ or from the configuration file. If a configuration file is specified, users should set paths for all input files and specify the analysis with corresponding parameters. Once file paths and parameters are set, the execution can be either run locally or scaled up to various high-performance computing environments.

### Output and Error Handling

Output files are automatically arranged in a nested folder structure (Fig. 1). Each lowest-level subfolder holds output and log files from executed process. In addition, Nextflow creates its own work folder to hold intermediate output files, which serves as a record of a run. If errors are encountered in a run, users can fix the specific error and re-run the pipeline with the “-resume” command. Once resumed, the pipeline automatically uses the cached files from the previous run and continues processes that have been fixed (Di Tommaso P et al., 2017).

## Conclusions and Discussions

The GWAS pipeline provides a user-friendly one-step analysis tool. The workflow balances flexibility and reproducibility by requiring limited options and parameters. To run more study-specific analyses, users may need to modify some parameters in the original scripts. While three types of analyses are currently available, the Nextflow-based pipeline is easy to extend with new tools and methods. We plan to include more complex models and analyses, such as survival analysis, in the next versions of the pipeline.

## Figures and Tables

**Figure 1:**